# Epidemiology of Invasive *Haemophilus influenzae* Disease, Europe, 2007–2014

**DOI:** 10.3201/eid2303.161552

**Published:** 2017-03

**Authors:** Robert Whittaker, Assimoula Economopoulou, Joana Gomes Dias, Elizabeth Bancroft, Miriam Ramliden, Lucia Pastore Celentano, European Centre for Disease Prevention and Control Country Experts for Invasive Haemophilus influenzae Disease

**Affiliations:** European Centre for Disease Prevention and Control, Solna, Sweden

**Keywords:** invasive *Haemophilus influenzae* disease, epidemiology, Europe, serotype, surveillance, coccobacillus, bacteria, invasive disease, vaccine, invasive pathogen, temporal trends

## Abstract

We describe the epidemiology of invasive *Haemophilus influenzae* disease during 2007–2014 in 12 European countries and assess overall *H.* *influenzae* disease trends by serotype and patient age. Mean annual notification rate was 0.6 cases/100,000 population, with an increasing annual trend of 3.3% (95% CI 2.3% to 4.3%). The notification rate was highest for patients <1 month of age (23.4 cases/100,000 population). Nontypeable *H.* *influenzae* (NTHi) caused 78% of all cases and showed increasing trends among persons <1 month and >20 years of age. Serotype f cases showed an increasing trend among persons >60 years of age. Serotype b cases showed decreasing trends among persons 1–5 months, 1–4 years, and >40 years of age. Sustained success of routine *H.* *influenzae* serotype b vaccination is evident. Surveillance systems must adopt a broad focus for invasive *H.* *influenzae* disease. Increasing reports of NTHi, particularly among neonates, highlight the potential benefit of a vaccine against NTHi.

*Haemophilus influenzae*, a pleomorphic gram-negative coccobacillus, is a common commensal of the upper respiratory tract. It is a human-only pathogen that can cause severe invasive disease, including meningitis, pneumonia, and septicemia. *H. influenzae* strains are divided based on the presence or absence of a polysaccharide capsule; there are 6 encapsulated serotypes (*H. influenzae* serotypes a [Hia], b [Hib], c [Hic], d [Hid], e [Hie], and f [Hif]) and nonencapsulated, nontypeable *H.* *influenzae* (NTHi) strains. Although Hib strains are considered the most pathogenic, NTHi accounts for a high proportion of all *H.* *influenzae* infections because it causes a notable number of noninvasive infections, such as otitis media and sinusitis, as well as invasive infections (*1*–*4*).

Beginning in 1989, countries of the European Union and European Economic Area (EU/EEA) began introducing conjugate Hib vaccination into their routine national immunization programs; most countries introduced the vaccine before the year 2000. In the prevaccine era, Hib was estimated to cause most cases of invasive *H.* *influenzae* disease and was a leading cause of bacterial meningitis worldwide, primarily among otherwise healthy children <5 years of age (*5*,*6*). The introduction of Hib vaccine has led to a substantial and sustained reduction in infection caused by Hib (*7*–*12*) and in pharyngeal Hib carriage, resulting in herd protection (*8*,*13*,*14*). The World Health Organization recommends the inclusion of Hib vaccination in all routine infant immunization programs as a 3-dose primary schedule with or without a booster dose or as a 2-dose primary schedule with a booster dose (*15*). Since 2010, Hib vaccination has been part of the national immunization program in all EU/EEA countries, and high coverage has been maintained (*16*). Following the introduction of Hib vaccine, several studies in Europe and elsewhere reported increasing trends in NTHi, Hia, Hie, and Hif infections (*3*,*4*,*7*,*17*,*18*), and NTHi is now the leading cause of invasive *H.* *influenzae* disease in EU/EEA countries and other areas worldwide (*2*–*4*). Most studies do not report evidence of strain replacement due to Hib vaccine introduction, although some have supported this occurrence (*7*–*9*,*11*,*17*,*19*–*21*).

In 1996, the European Union Invasive Bacterial Infections Surveillance Network began Europe-level surveillance of invasive *H.* *influenzae* disease, and since 2007, surveillance has been coordinated by the European Centre for Disease Prevention and Control (ECDC) (*7*). We conducted a study to describe the epidemiology of invasive *H.* *influenzae* disease in EU/EEA countries during 2007–2014 and to monitor age- and serotype-specific trends during the study period.

## Methods

### European Surveillance of Invasive *H.* *influenzae* Disease

On an annual basis, all 28 EU Member States and 2 EEA countries report national surveillance data on invasive *H.* *influenzae* disease to a central database at ECDC. Most of the 30 reporting countries provide data from passive surveillance systems, including mandatory reporting, that cover their entire national populations (*22*). All 30 countries report using the EU case definition for invasive *H. influenzae* (*23*) or a case definition with compatible criteria for laboratory confirmation of disease. Invasive *H.* *influenzae* disease is confirmed by isolation of *H.* *influenzae* from a normally sterile site; culture is used for confirmation of >99% of all reported cases. According to the most recent external quality assurance scheme run by the ECDC-funded IBD-labnet (the invasive bacterial disease laboratory surveillance network in Europe), 20 countries also use a PCR-based method to confirm species identity. Twenty-eight countries routinely serotype isolates, most by slide agglutination, PCR, or both methods (*24*).

### Data Selection and Preparation

We analyzed data on invasive *H.* *influenzae* disease reported to ECDC during 2007–2014. We excluded cases not reported as laboratory-confirmed or for patients with unreported age or sex. We excluded data from countries that 1) had not reported case-based data for all years in the study period; 2) had introduced Hib vaccination into their national immunization program during the study period; 3) had reported >50% of cases as meningitis, Hib, or both, which may indicate a surveillance bias toward the reporting of these cases; or 4) had not reported serotype data for all years and/or had reported serotype data for <50% of cases.

We used surveillance system coverage data and population data from Eurostat (http://www.ec.europa.eu/eurostat) as denominators for calculating the total and age-specific notification rates per 100,000 population. We categorized data on age into the following patient age groups; <1, 1–4, 5–19, 20–39, 40–59, and >60 years of age. We further categorized the infant (<1 year of age) age group into <1 month, 1–5 months, and 6–11 months of age. We estimated the denominator in these infant age groups as the total infant population divided by 12 and multiplied by the number of months in each age group. Countries that did not report data on the age of infants in months were excluded from the analysis of infant age groups.

### Data Analysis

We described the epidemiology of invasive *H.* *influenzae* disease by year, country, and serotype and by patient age group, sex, and clinical presentation. We compared patient age distributions by *H.* *influenzae* serotype by calculating median ages with interquartile ranges and comparing them using the Kruskal-Wallis test. The Dunn test was used to perform post hoc pairwise multiple comparisons. We used male:female notification rate ratios to describe the sex distribution of patients by age group, serotype, or both. We applied Poisson regression models to estimate differences in male and female notification rates and male:female notification rate ratios. We expressed categorical variables as the number of cases and proportion (%) and compared them using the χ^2^ test.

We assessed overall temporal trends by estimating the percentage change in annual notification rates, including 95% CIs, by age group, serotype, or both by using linear regression analysis of the log of the annual notification rate. We used reporting country as a cluster effect in the models. We fixed the significance level at p = 0.05 and used Stata 14 (StataCorp LLC, College Station, TX, USA) to analyze data.

## Results

We included data from 12 of the 30 EU/EEA countries: Belgium, Cyprus, the Czech Republic, Denmark, Finland, Ireland, Italy, the Netherlands, Norway, Slovenia, Spain, and the United Kingdom. Belgium and Spain had voluntary reporting, but the other countries had mandatory reporting. Belgium and the Czech Republic described their surveillance system as active; all other countries reported having passive surveillance systems. Surveillance system population coverage was 50% in Spain and 100% in the other 11 countries. Together, the surveillance systems in these 12 countries covered 41% of the total EU/EEA population. The year of Hib vaccine introduction in the 12 countries ranged from 1992 to 2001. With 1 exception, 3-dose vaccination coverage was >90% in all countries during the study period; Denmark had 87%–89% coverage during 2007–2009 (*16*).

Of the remaining 18 EU/EEA countries, we excluded 4 for not reporting case-based data for all study years (Bulgaria, Croatia, Luxembourg, Romania) and 2 for introducing the vaccine during the study period (Bulgaria, Poland). We also excluded 5 countries for reporting >50% of cases as meningitis or Hib (Estonia, Greece, Hungary, Latvia, Slovakia), and we excluded 8 for not reporting serotype data for all years, reporting serotype data for <50% of cases, or both (Austria, France, Germany, Iceland, Lithuania, Malta, Portugal, Sweden).

**Figure 1:**
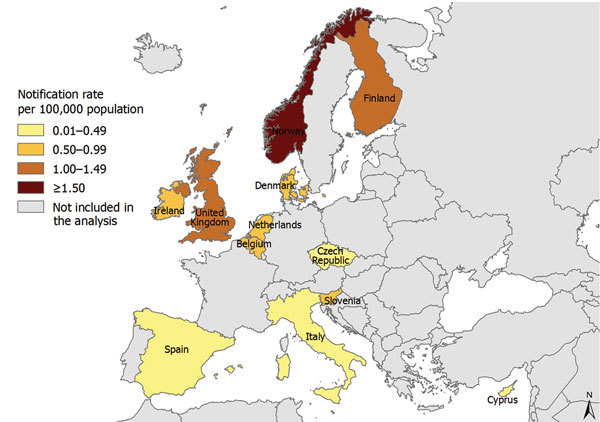
Notification rate for cases of invasive of *Haemophilus influenzae* disease in 12 European countries, 2007–2014. A total of 10,624 cases were notified.

During 2007–2014, the 12 countries included in the study reported a total of 10,624 cases of invasive *H.* *influenzae* disease for a mean annual notification rate of 0.6 cases/100,000 population. The overall notification rate increased 3.3% (95% CI 2.3% to 4.3%) annually during the study period (Table 1). By country, the notification rate ranged from 1.6 cases/100,000 population (n = 637) in Norway to 0.1 case/100,000 population (n = 6) in Cyprus (Figure 1). We observed increasing overall trends in Denmark, Italy, the Netherlands, and Spain and insignificant trends in all other countries.

**Table 1:** Epidemiologic findings for cases of invasive *Haemophilus influenzae* disease, by patient age group and year of notification, in 12 countries in Europe, 2007–2014*

Age group	Annual notification rate/100,000 population (no. cases)								Mean annual notification rate (no. cases)	M:F ratio	% Change in annual notification rate (95% CI)†
	2007	2008	2009	2010	2011	2012	2013	2014			
<1 y	4.3 (96)	4.4 (101)	5.4 (125)	4.4 (103)	5.2 (120)	4.7 (106)	4.3 (97)	6.5 (140)	4.9 (888)	1.24	2.8 (−2.1 to 8.0)
<1 mo‡	17.5 (29)	21.3 (36)	24.8 (43)	23.2 (40)	25.5 (44)	20.0 (34)	24.3 (41)	30.8 (51)	23.4 (318)	1.04	5.0 (−0.2 to 10.4)
1–5 mo‡	4.5 (37)	3.3 (28)	3.8 (33)	3.0 (26)	3.4 (29)	2.9 (25)	1.9 (16)	3.0 (25)	3.2 (219)	1.75	−7.1 (−13.3 to 0.4)
6–11 mo‡	2.3 (23)	3.0 (30)	3.6 (37)	1.8 (19)	2.7 (28)	3.3 (34)	2.8 (28)	4.5 (45)	3.0 (244)	1.16	6.0 (−4.3 to 16.5)
1–4 y	1.1 (97)	0.9 (80)	1.0 (88)	0.7 (67)	0.9 (80)	0.7 (62)	0.9 (85)	1.0 (93)	0.9 (652)	1.25	−2.01 (−8.3 to 4.6)
5–19 y	0.2 (58)	0.2 (63)	0.2 (61)	0.1 (45)	0.2 (63)	0.1 (44)	0.2 (57)	0.2 (71)	0.2 (462)	1.29	0.3 (−6.3 to 7.3)
20–39 y	0.2 (113)	0.2 (118)	0.2 (111)	0.2 (134)	0.3 (139)	0.2 (114)	0.2 (115)	0.3 (148)	0.2 (992)	0.56	2.8 (−1.2 to 6.9)
40–59 y	0.3 (190)	0.4 (204)	0.4 (209)	0.4 (201)	0.4 (255)	0.4 (206)	0.40 (228)	0.4 (230)	0.4 (1,723)	0.98	1.5 (−1.4 to 4.6)
>60 y	1.3 (606)	1.4 (638)	1.4 (675)	1.5 (703)	1.5 (727)	1.7 (848)	1.7 (836)	1.7 (874)	1.5 (5,907)	1.28	**3.8 (2.5 to 5.1)**
Total§	0.6 (1,160)	0.6 (1,204)	0.6 (1,269)	0.6 (1,253)	0.7 (1,384)	0.7 (1,380)	0.7 (1,418)	0.7 (1,556)	0.6 (10,624)	1.05	**3.3 (2.3 to 4.3)**
*The study was conducted in Belgium, Cyprus, the Czech Republic, Denmark, Finland, Ireland, Italy, the Netherlands, Norway, Slovenia, Spain and the United Kingdom. Data are for a total of 10,624 cases. †Bold font indicates statistically significant trends (p = 0.05). ‡For these age groups, data from only 11 countries are included because Spain did not report data on the age of infant cases by month. §Totals do not include data separately shown for infants <1 mo, 1–5 mo, and 6–11 mo of age because those data are included in the <1 y age group.											

### Age and Sex of Case-Patients

Of the 10,624 case-patients, 5,907 (56%) were >60 years of age, and 888 (8.4%) were <1 year of age (Table 1). The notification rate was highest for infants (4.9 cases/100,000 population), followed by persons >60 years of age (1.5/100,000). The notification rate among persons >60 years of age increased 3.8% (95% CI 2.5% to 5.1%) annually. The age in months was available for 781 (88%) of the 888 infants; Spain was the only country not to report any data on month of age. The notification rate for infants <1 month of age (23.4 cases/100,000 population) was >7-fold higher than that for those 1–5 months of age (3.2/100,000) and 6–11 months of age (3.0/100,000). The notification rate for infants 1–5 months of age decreased 7.1% (95% CI −13.3% to −0.4%) annually. The overall male:female notification rate ratio was 1.05 (95% CI 1.01 to 1.09) (Table 1).

### Serotype

*H.* *influenzae* serotype was reported for 8,781 (83%) of the 10,624 patients (Table 2). The age distribution did not differ between case-patients with serotype reported and those with serotype not reported (p = 0.319). Case-patients without a reported serotype were more likely than those with a reported serotype to be male (male:female notification rate ratio 1.20 vs. 0.97, respectively; p = 0.001).

**Table 2:** Mean annual notification rate per 100,000 population and number of cases of invasive *Haemophilus influenzae* disease by serotype and patient age group, in 12 European countries, 2007–2014*

Variable	Mean annual notification rate/100,000 population, by serotype (no. cases)†										Mean overall annual notification rate (no. cases)
	Hia	Hib	Hic	Hid	Hie	Hif	NTHi	Non-b	Total with data	Unknown	
Age group											
<1 y	0.05 (9)	0.65 (118)	0	0	0.06 (11)	0.30 (54)	3.17 (578)	0.005 (1)	4.24 (771)	0.64 (117)	4.90 (888)
<1 mo‡	0.07 (1)	0.29 (4)	0	0	0 (0)	0.22 (3)	19.37 (263)	0	19.96 (271)	3.46 (47)	23.42 (318)
1–5 mo‡	0.01 (1)	0.65 (44)	0	0	0.06 (4)	0.22 (15)	1.75 (119)	0.01 (1)	2.71 (184)	0.52 (35)	3.23 (219)
6–11 mo‡	0.09 (7)	0.74 (60)	0	0	0.09 (7)	0.33 (27)	1.44 (117)	0	2.68 (218)	0.32 (26)	3.00 (244)
1–4 y	0.006 (4)	0.18 (132)	0.001 (1)	0.003 (2)	0.01 (8)	0.07 (52)	0.51 (369)	0.006 (4)	0.79 (572)	0.11 (80)	0.90 (652)
5–19 y	<0.001 (1)	0.02 (54)	<0.001 (1)	0	0.003 (9)	0.01 (26)	0.10 (277)	<0.001 (1)	0.14 (369)	0.03 (93)	0.17 (462)
20–39 y	<0.001 (1)	0.02 (71)	0	<0.001 (1)	0.003 (12)	0.01 (50)	0.15 (648)	<0.001 (2)	0.18 (785)	0.05 (207)	0.22 (992)
40–59 y	<0.001 (4)	0.04 (189)	0	<0.001 (1)	0.009 (41)	0.03 (153)	0.21 (980)	<0.001 (1)	0.30 (1,369)	0.08 (354)	0.37 (1,723)
>60 y	0.001 (4)	0.06 (247)	0	<0.001 (6)	0.04 (158)	0.13 (493)	1.03 (4,001)	<0.001 (6)	1.27 (4,915)	0.26 (992)	1.53 (5,907)
Overall notification rate (no. cases)§	0.001 (23)	0.05 (811)	<0.001 (2)	<0.001 (10)	0.01 (239)	0.05 (828)	0.42 (6,853)	<0.001 (15)	0.53 (8,781)	0.11 (1,843)	0.64 (10,624)
Median age, y§	2	43	3	69	66	64	65	32	64	62	63
IQR, y§	0–55	3–63	1–5	33–76	53–78	45–75	35–79	2–83	33–78	37–76	34–77
*The study was conducted in Belgium, Cyprus, the Czech Republic, Denmark, Finland, Ireland, Italy, the Netherlands, Norway, Slovenia, Spain, and the United Kingdom. Data are for a total of 10,624 cases. Hia, *H. influenzae* serotype a; Hib, serotype b; Hic, serotype c; Hid, serotype d; Hie, serotype e; Hif, serotype f; NTHi, nontypeable *H. influenzae*; non-b, cases reported as a non-b *H. influenzae* strain (It was not known whether these cases were encapsulated); IQR, interquartile range. †Values are mean annual notification rate/100,000 population (no. cases), except as indicated. ‡For these age groups, data from only 11 countries are included because Spain did not report data on the age of infant cases by month. §Totals do not include data separately shown for infants <1 mo, 1–5 mo, and 6–11 mo of age because those data are included in the <1 y patient age group.											

A total of 6,853 (78%) of the 8,781 cases with a reported serotype were caused by NTHi strains; these strains also accounted for most cases in all age groups (Table 2). The notification rate for NTHi cases was highest among infants and persons >60 years of age; most cases were in the older age group. We observed this same notification profile among Hie (239/8,781 [3%]) and Hif (828/8,781 [9%]) cases. Case-patients with Hib infection (811/8,781 [9%]) had a lower median age than those with Hie (p<0.001), Hif (p<0.001), or NTHi (p<0.001) infection. Hib caused 19% (250/1,343) of all cases among children <5 years of age and had highest notification rates among infants and children 1–4 years of age. However, most Hib cases were in persons >40 years of age (Table 2). *H. influenzae* serotype was reported for 86% (673/781) of infants with known month of age. NTHi caused most cases in all infant age groups; most notably, NTHi caused 97% (263/271) of cases among infants <1 month of age (a notification rate of 19.4 cases/100,000 population) (Table 2).

Among 20- to 39-year-old patients, more women than men were infected with Hie (male:female notification rate ratio 0.09, 95% CI 0.11 to 0.69), Hif (0.55, 95% CI 0.31 to 0.99), and NTHi (0.44, 95% CI 0.38 to 0.53). Conversely, among patients >60 years of age, more men than women were infected by Hie (1.45, 95% CI 1.06 to 1.99) and NTHi (1.30, 95% CI 1.22 to 1.38), and more boys than girls were infected by NTHi among children <1 year of age (1.20, 95% CI 1.02 to 1.42) and 1–4 years of age (1.37, 95% CI 1.11 to 1.69).

**Figure 2:**
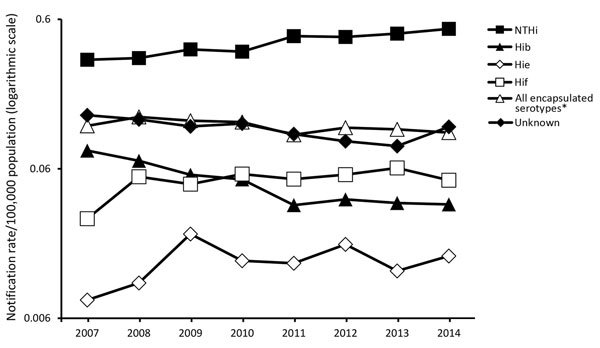
Notification rate for cases of invasive *Haemophilus influenzae* disease, by serotype and year of notification, in 12 countries in Europe, 2007–2014. A total of 8,781 cases were notified. Cases were notified from Belgium, Cyprus, the Czech Republic, Denmark, Finland, Ireland, Italy, the Netherlands, Norway, Slovenia, Spain, and the United Kingdom. *Refers to all cases reported as *H. influenzae* serotypes a (Hia), b (Hib), c (Hic), d (Hid), e (Hie), and f (Hif).

The notification rate of NTHi cases increased 7.4% (95% CI 5.3% to 9.6%) annually, driven by increasing trends in NTHi cases among children <1 year of age and persons >20 years of age. The increasing trend in infants was driven by a 6.2% (95% CI 2.8% to 9.8%) annual increase in the notification rate among those <1 month of age (Table 3). The notification rate of Hib cases decreased 11.9% (95% CI −16.0% to −7.5%) annually, driven by decreasing trends in Hib cases among persons <1 year, 1–4 years, 40–59 years, and <60 years of age (Figure 2; Table 3). The decreasing trend in infants was driven by a 25.0% (95% CI −32.2% to −17.0%) annual decrease in cases among infants 1–5 months of age (Table 3). No significant overall trend was observed among Hie or Hif cases or collectively among cases caused by encapsulated serotypes Hia–Hif (Figure 2; Table 3). The notification rate of Hie cases among children 1–4 years of age decreased 14.2% (95% CI −25.0% to −1.7%) annually (Table 3), although only 8 cases were reported for this serotype and age group during the study period (Table 2). The notification rate of Hif cases among persons >60 years of age increased 7.0% (95% CI 0.9% to 13.4%) annually (Table 3). Each year during 2010–2014, more cases of Hif than Hib were reported (Figure 2). Too few cases of Hia, Hic, and Hid were reported to calculate trends for these serotypes (Table 2). The notification rate of cases reported with unknown serotype decreased 4.8% (95% CI −9.0% to −0.5%) annually (Figure 2; Table 3).

**Table 3:** Percentage change in annual notification rate for cases of invasive *Haemophilus influenzae* disease, by serotype and patient age group, in 12 European countries, 2007–2014*

Age group	% Change (95% CI), N = 10,574†				
	Hib	Hie	Hif	NTHi	Unknown serotype
<1 y	**−8.5 (−14.5 to −2.1)**	−4.3 (−33.6 to 38.1)	−6.2 (−23.6 to 15.2)	**5.5 (0.6 to 10.8)**	4.1 (−4.1 to 13.0)
<1 mo‡	–	–	−0.9 (−4.3 to 2.6)	**6.2 (2.8 to 9.8)**	−0.3 (−19.3 to 23.1)
1–5 mo‡	**−25.0 (−32.2 to −17.0)**	−2.4 (−72.9 to 251.8)	11.7 (−8.2 to 36.0)	2.1 (−3.3 to 7.7)	−7.1 (−15.6 to 2.4)
6–11 mo‡	3.5 (−18.4 to 31.1)	–	−4.8 (−21.2 to 15.1)	2.7 (−9.7 to 16.8)	24.9 (−2.9 to 60.7)
1–4 y	**−18.4 (−32.9 to −0.8)**	**−14.2 (−25.0 to −1.7)**	10.1 (−8.7 to 32.7)	3.8 (−3.4 to 11.6)	2.8 (−9.3 to 16.5)
5–19 y	−8.3 (−26.2 to 14.1)	–	−0.2 (−26.3 to 35.0)	5.3 (−4.7 to 16.3)	−4.9 (−20.3 to 13.5)
20–39 y	−15.0 (−29.4 to 2.3)	3.4 (−14.8 to 25.5)	−1.4 (−17.6 to 18.0)	**9.7 (5.6 to 13.9)**	**−8.6 (−16.2 to −0.3)**
40–59 y	**−9.0 (−14.7 to −3.0)**	−3.3 (−19.8 to 16.7)	7.0 (−3.8 to 19.0)	**6.8 (2.4 to 11.3)**	**−6.8 (−11.0 to −2.3)**
>60 y	**−12.6 (−17.8 to −7.1)**	12.7 (−2.9 to 30.8)	**7.0 (0.9 to 13.4)**	**7.0 (4.5 to 9.5)**	−6.0 (−12.1 to 0.5)
Total§	**−11.9 (−16.0 to −7.5)**	6.3 (−5.3 to 19.5)	6.4 (−1.5 to 14.8)	**7.4 (5.3 to 9.6)**	**−4.8 (−9.0 to −0.5)**
*The study was conducted in Belgium, Cyprus, the Czech Republic, Denmark, Finland, Ireland, Italy, the Netherlands, Norway, Slovenia, Spain, and the United Kingdom. Data are for a total of 10,574 cases. Hib, *H. influenzae* serotype b; Hie, serotype e; Hif, serotype f; NTHi, nontypeable *H. influenzae*; – no cases reported or no trend could be determined. †Bold font indicates statistically significant trends (p = 0.05). ‡For these age groups, data from only 11 countries are included because Spain did not report data on the age of infant cases by month. §Totals do not include data separately shown for infants <1 mo, 1–5 mo, and 6–11 mo of age because those data are included in the <1 y age group.					

By country, an 18.5% (95% CI 1.9% to 37.9%) increasing trend in Hib was observed in Italy, although only 26 cases were reported during the study period, and no more than 5 cases were reported in a single year. The notification rate did not increase significantly for any encapsulated serotype in any other country. The notification rate for NTHi cases increased significantly in Belgium, Denmark, Ireland, Italy, the Netherlands, Spain, and the United Kingdom (data not shown). In all other countries, the change in the NTHi notification rate over the study period was not significant.

### Clinical Presentation

Clinical presentation was known for 6,722 (63%) of the reported 10,624 case-patients. Most had septicemia (4,128 patients [61%]), bacterial pneumonia (1,207 [18%]), or meningitis (596 [9%]). The following clinical presentations were also reported: osteomyelitis (75 patients [1%]), meningitis and septicemia (64 [1%]), epiglottitis (52 [1%]), and cellulitis (37 [1%]), and other (563 [8%]). Septicemia was the most common clinical presentation in all age groups.

**Figure 3:**
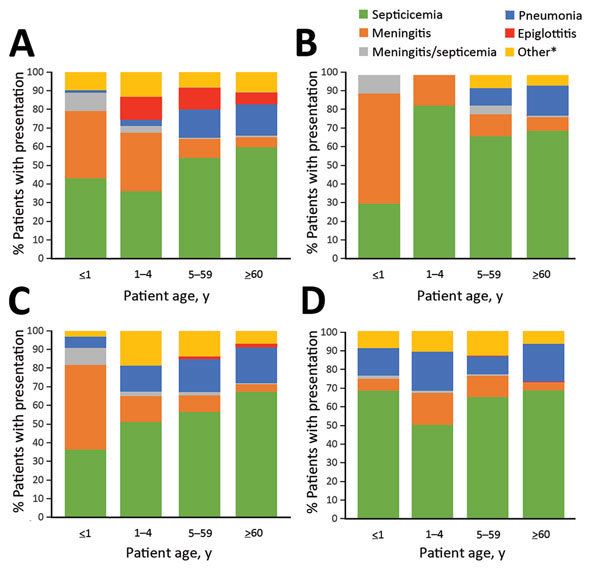
Percentage of cases, by patient age group, in 12 countries in Europe with various clinical presentations of *Haemophilus influenzae* disease caused by serotypes b (A), e (B), and f (C) and by nontypeable *H. influenzae* (D), 2007–2014. Cases (N = 5,879) were in Belgium, Cyprus, the Czech Republic, Denmark, Finland, Ireland, Italy, the Netherlands, Norway, Slovenia, Spain, and the United Kingdom. *Refers to cases reported as other, cellulitis, septic arthritis, or osteomyelitis.

Clinical presentation was known for 5,913 (67%) of the 8,781 patients with serotyped isolates. For all the different clinical presentations, except epiglottitis, NTHi was the most common cause of *H. influenzae* infection; 78% of cases presenting with epiglottitis were caused by Hib. Septicemia was reported for most cases caused by Hib (51%), Hie (67%), Hif (61%), and NTHi (66%), and it was the most common clinical presentation for all age groups infected with these serotypes, except infants infected with Hie and Hif (60% and 45%, respectively, were reported to have meningitis) (Figure 3). Bacterial pneumonia was most prominent among older age groups with Hib, Hie, and Hif infection, but it was observed across all age groups with NTHi infection (Figure 3). Among 212 infants <1 month of age with available clinical presentation and serotype data, 181 (85%) had NTHi infection presenting with septicemia.

## Discussion

The sustained low notification rate for Hib and continued decreasing infection trend in all age groups (i.e., in those targeted and not targeted for vaccination) underscore the success of routine Hib vaccination. Among children <5 years of age with invasive *H. influenzae* disease, almost 1 in 5 cases is still caused by Hib, a potentially preventable disease. Breakthrough cases of invasive disease following Hib vaccination have been reported in immunocompromised and healthy children (*25*,*26*); however, vaccine failures are rare, and additional vaccine doses have are an effective way to achieve protective antibody levels in such instances (*25*). Although Hib vaccination has notably decreased the incidence of invasive Hib disease in all age groups, this reduction has been greatest among young children (*3*,*10*,*11*,*27*), and most Hib cases now occur in older adults with concurrent conditions (*27*,*28*).

In the prevaccine era, NTHi was not a known common cause of invasive infection (*29*), but it is now well recognized as the leading cause of invasive *H.* *influenzae* disease (*2*–*4*). Higher *H.* *influenzae* notification rates for infants, particularly neonates, the elderly, and women of childbearing age, were described before (*30*,*31*) and after (*32*–*34*) the introduction of routine Hib vaccination. In addition, several studies showed an increased burden of NTHi in groups more susceptible to infection, with high proportions of intensive care admission, high case-fatality rates, and frequent sequelae among survivors (*2*,*29*,*32*,*35*). The notification rate of NTHi cases in infants <1 month of age, with most cases presenting as septicemia, is particularly striking. Studies have shown that most cases in neonates are present at the time of birth, and infection may induce labor (*33*), causing premature birth (*3*,*33*,*36*). It is probable that the number of NTHi infections among neonates is underestimated (*37*), although the increasing notification rate among infants <1 month of age indicates that reporting may be improving. If developed, a vaccine against NTHi that could be administered to pregnant women could provide protection to expectant mothers and neonates (*35*). The genetic diversity of NTHi complicates vaccine development, but exploration into potential NTHi vaccine candidates is ongoing (*38*).

The increasing recognition of NTHi as a key invasive pathogen highlights how future surveillance of invasive *H.* *influenzae* disease must encompass all serotypes and strains, age groups, and clinical presentations. EU/EEA member states are not required to report all *H.* *influenzae* strains. Moreover, simply studying NTHi trends may now be insufficient for monitoring changes in the epidemiology of NTHi strains because they are more genetically diverse than encapsulated strains (*29*,*35*,*37*,*39*). Surveillance of NTHi in Europe may benefit from more genetic typing studies of circulating strains, with regard to carriage and disease, and the standardization of typing methodologies (*24*,*36*,*37*).

The notification rate of non-Hib encapsulated serotypes in Europe remains low and stable. Some studies have reported increasing trends in Hia cases after the introduction of routine Hib vaccination (*18*,*40*,*41*); however, Hia remains rare in Europe.

We observed increasing trends in the annual notification rate of NTHi cases in persons <1 and >20 years of age and of Hif cases in persons >60 years of age. These trends may represent an actual increase in the incidence of disease, which could result from different factors, such as population aging and increased use of immunosuppressive therapy, both of which would increase the number of persons at risk for infection by these strains (*17*,*35*). Despite these increasing trends, we could not assess possible strain replacement resulting from the introduction of Hib vaccination because we could not compare serotype distributions or incidence between the prevaccination and postvaccination periods. Trends also may reflect changes and improvements in surveillance that increase case detection, such as an increase in awareness among clinicians since Hib vaccine introduction, changing blood culture practices, and more accurate serotyping techniques. For example, since 1993 in the Netherlands, the reporting of NTHi from blood isolates has increased, while the reporting of NTHi from cerebrospinal fluid isolates has remained stable (*42*). Furthermore, new molecular technologies, such as PCR-based serotyping, have allowed more accurate differentiation between typeable and nontypeable strains (*24*,*37*). Such technologies are becoming more widely used across the EU/EEA; in 2014, a total of 24 reference laboratories performed PCR-based serotyping, compared with 19 laboratories in 2012 (*24*).

Limitations of our study were the need to combine and compare data from different countries that had possible differences in surveillance sensitivity and methodology and the predisposition for underreporting in routine passive surveillance systems (*43*). The notification rate of invasive *H.* *influenzae* disease in the United States in 2014, detected through Active Bacterial Core surveillance, was >2 times that of the 12 countries in this study (*44*). Nevertheless, for the entire study period, all included countries used comparable case definitions and reported consistently high quality data for all age groups, serotypes, and clinical presentations, thus indicating no potential surveillance bias. Together, these 12 countries covered 41% of the EU/EEA population, higher than the population coverage in similar large studies (*3*,*7*), and trends observed in each country were consistent with the pooled results for Europe. The surveillance of invasive *H.* *influenzae* disease on the Europe level is longstanding (*7*) and allows the pooling of data to increase the precision of estimates for what is now a rare disease in the EU/EEA. National reference laboratories in all countries participate in the external quality assurance schemes and training run by IBD-labnet (*24*). Unfortunately, we could not assess specific risk factors, such as concurrent conditions, or sequelae among surviving case-patients because such data are not collected at ECDC. We also could not assess potential vaccine failures because the date of last vaccination was not collected for patients, and the completeness of data regarding the vaccination status of patients with Hib infection was low. In addition, data on fatal outcome were not included because completeness of the data was low. These limitations, along with the fact that data from only 12 of 30 countries were included, underscore the potential for improving the scope and quality of data reported to ECDC and increasing the value of surveillance on the Europe level.

In conclusion, the sustained success of routine Hib vaccination is evident, however the epidemiology of invasive *H.* *influenzae* disease must continue to be carefully monitored through surveillance systems with a broad focus. In addition, the continually increasing reporting of invasive disease caused by NTHi, particularly among neonates, highlights the potential benefit of the development of a vaccine against NTHi.

List of European Centre for Disease Prevention and Control Country Experts for Invasive *Haemophilus influenzae* Disease: Georg Steindl (Austria), Delphine Martiny and Tine Grammens (Belgium), Teodora Georgieva (Bulgaria), Panayiota Maikanti-Charalampous and Maria Koliou (Cyprus), Vera Lebedova and Pavla Krizova (Czech Republic), Tine Dalby and Palle Valentiner-Branth (Denmark), Jevgenia Epstein and Natalia Kerbo (Estonia), Maija Toropainen and Markku Kuusi (Finland), Scarlett Georges and Agnès Lepoutre (France), Anja Takla and Thien-Tri Lam (Germany), Theano Georgakopoulou and Georgina Tzanakaki (Greece), Zsuzsanna Molnár (Hungary), Thorolfur Gudnason and Hjordis Hardardottir (Iceland), Piaras O’Lorcain and Kenneth Meyler (Ireland), Fortunato D'Ancona and Marina Cerquetti (Italy), Larisa Savrasova and Jelena Galajeva (Latvia), Greta Gargasienė (Lithuania), Paul Caruana and Tanya Melillo (Malta), Liesbeth Mollema and Lodewijk Spanjaard (Netherlands), Martin Steinbakk (Norway), Iwona Paradowska-Stankiewicz and Alicja Kuch (Poland), Cátia Sousa Pinto and Paula Lavado (Portugal), Aurora Stanescu and Mihaela Cristina Giuca (Romania), Elena Novakova (Slovakia), Tamara Kastrin and Marta Grgic Vitek (Slovenia), Maria Pérez-Vázquez and Rosa Cano (Spain), Tiia Lepp and Eva Morfeldt (Sweden), and Shamez Ladhani and Eisin McDonald (United Kingdom).
